# Assessment of long-read strategies for the enrichment of clinically relevant breakpoints in lymphomas: towards a diagnostic implementation

**DOI:** 10.1007/s00277-026-06754-2

**Published:** 2026-01-21

**Authors:** Filip Pardy, Kamila Reblova, Hana Svozilova, Boris Tichy, Sarka Pospisilova, Jana Kotaskova, Veronika Navrkalova

**Affiliations:** 1 Centre of Molecular Biology and Genetics, Department of Internal Medicine – Hematology and Oncology, University Hospital Brno and Faculty of Medicine, Masaryk University, Brno, Czech Republic; 2 Center of Molecular Medicine, CEITEC - Central European Institute of Technology, Masaryk University, Brno, Czech Republic; 3 Institute of Medical Genetics and Genomics, Faculty of Medicine, Masaryk University and University Hospital Brno, Brno, Czech Republic

**Keywords:** Oxford Nanopore sequencing, Target enrichment, Translocations, Lymphoma, Molecular diagnostics

## Abstract

Recurrent chromosomal translocations are hallmarks of many hematological malignancies, including lymphomas and leukemias. Accurate breakpoint detection is essential for diagnostics, treatment optimization, and disease monitoring. Long-read sequencing (Oxford Nanopore Technologies) enables unambiguous mapping and translocation identification. We designed a Cas9-based enrichment panel targeting common translocations in lymphoid malignancies. To accommodate both well-defined and promiscuous translocation partners, we employed single-side and dual-side sequencing strategies. Using well-established lymphoid cell lines, we benchmarked three enrichment approaches: (i) Cas9 read-out, (ii) Cas9 excision with multiplexing, and (iii) adaptive sampling. Cas9-mediated enrichment achieved superior on-target coverage, particularly in densely targeted regions (such as the IGH locus), while single-probe targets showed lower coverage depth. Adaptive sampling offered higher throughput, flexibility, and better pore occupancy, however with limited breakpoint detection. Cas9 excision has been demonstrated as a fast and reliable method to detect canonical translocation partners in clinical lymphoma samples. Our findings indicate that long-read enrichment strategies are suitable for targeting breakpoint hotspots, although the choice of approach depends heavily on the laboratory's specific goal. We propose a decision algorithm for selecting the optimal method based on experimental and clinical needs: Cas9-mediated enrichment suits focused diagnostic intent, while adaptive sampling is preferable for broader research use.

**Supplementary Information:**

The online version contains supplementary material available at 10.1007/s00277-026-06754-2.

## Introduction

Structural variants (SVs) are one of the major sources of variability in the human genome and a cause of multiple hereditary and malignant diseases [1, 2]. The functional significance of such rearrangement can vary [3, 4]. The translocation can lead to generating a fusion gene with a functional transcript. One of the best described examples is the t(9;22) involving *BCR* and *ABL1* genes, forming a new functional fusion kinase that typically drives the proliferation of chronic myeloid leukemia cells. Other events can lead to the fusion of a strong promoter that enhances the transcription of a less active gene. Lastly, transcriptional activation can happen when the regulatory region is juxtaposed in proximity to a protooncogene, thereby causing gene overexpression [5]. Such oncogenic lymphoma translocation often involves genes like *MYC*, *BCL2*, *BCL6*, or *CCND1*. Up to date, more than 34,000 unique gene fusions have been listed in the Mitelman database [6].

Accurate detection of breakpoint regions is relevant for diagnostics and patient monitoring in hemato-oncology practice. The variability in size and genetic context of structural variations poses a technical challenge for their detection. Traditional methods such as fluorescence *in situ* hybridization (FISH) or short-read sequencing (NGS) are limited in the detection of complex SVs and unknown partners [7, 8]. Immunohistochemistry, as a surrogate test of affected gene overexpression, cannot fully replace direct genetic analysis [9]. Long-read sequencing technologies such as Oxford Nanopore Technologies (ONT) overcome these limitations by enabling comprehensive mapping of extensive genomic rearrangements within single reads.

Specifically, breakpoint regions in non-Hodgkin lymphomas might be very extensively spread, as shown in the case of follicular lymphoma and diffuse large B-cell lymphoma. While both diagnoses present translocations of the immunoglobulin heavy chain (IGH) locus, the exact breakpoint of the aberration might involve either the S*μ* or the S*γ* constant switch regions, which are hundreds of kilobases apart [10]. The occurrence of distinct breakpoints can be explained by different mechanisms, either by RAG-mediated translocation during the V(D)J recombination, or AID-mediated translocation during somatic hypermutation in post-GC lineages [11]. Due to the complex genetic context of the DNA sequence in the intronic regions of IGH and the large breakpoint area, it is often impossible to reliably align the short sequencing reads generated by other sequencing platforms. ONT sequencing can reach several hundred kilobases, effectively reading even through the repetitive regions.

Given the limited range of genetic translocations with clear clinical impact in lymphoid malignancies, the use of long-read whole genome sequencing (WGS) would be superfluous. Therefore, a targeting strategy that would interrogate particular gene loci would be more sensible, ideally in a partner-agnostic manner. Such enrichment strategies have already been described, especially the attractive approach based on the CRISPR/Cas9 cleavage in a directional fashion [12]. The other possible target enrichment option is the ONT's proprietary adaptive sampling (AS), which leverages the platform's real-time base calling and voltage reversal properties. In this regime, ONT software stalls the sequencing of a library strand after approximately 400 bp and aligns the generated read to the reference genome. Based on predefined genomic regions by the user, the strand is either accepted and sequenced in full length or rejected. Advantages were demonstrated in studies on pediatric acute leukemias by the rapid detection of key translocations with uniform coverage across target regions [13, 14].

In this study, we aimed to evaluate the Cas9 and AS enrichment strategies, with the possibility of sample multiplexing, and compare the benefits for diagnostics. We propose a clinical decision algorithm to balance sensitivity, flexibility, and turnaround time for individual laboratory needs. We demonstrate that integrating long-read sequencing with optimized enrichment strategies paves the way for personalized detection of translocation hotspots in routine diagnostics.

## Materials and methods

### Cell lines

Cell lines DOHH-2, Maver-1, Ramos, JVM-2, NALM-6, ML-2, Reh, Granta-452, and KCL-22, stored at the laboratory archive or subsequently obtained, were used in our study. Cells were cultivated for two weeks in RPMI-1640 medium supplemented with 10% fetal bovine serum (Biosera, France) previously heat-inactivated at 56 °C for 30 min. Following the culture, the genomic DNA was isolated using the MagCore instrument (RBC Bioscience, Taiwan). The quality and quantity of the isolated DNA were measured by Quantus fluorometer (Promega, WI, USA), NanoDrop 2000c (ThermoFisher Scientific, DE, USA), and Agilent Tapestation (Agilent, CA, USA) using the Genomic DNA ScreenTape and reagents.

### CRISPR/Cas9 probe set design

Cas9 probes were designed in the genomic regions of rearrangement hotspots in lymphoid malignancies. We used the interactive IDT designer at the IDT website with the "design custom gRNA" tool [15]. After the identification of regions of interest in the Ensembl genome browser, we exported 1 kb sequences that were then evaluated for the best-matching probes. Probes of the best specificity and lowest off-target scores were selected. We focused on lymphoma-specific translocation regions [16] by designing probes that flanked both partners of a typical translocation hotspot, e.g., *IGH::MYC*, *IGH::BCL6*, *IGH::BCL2*, and *IGH::CCND1*. We expanded the design with regions typically translocated in B-ALL, including *IGH, KMT2A, BCR,* and *RUNX1*, where probes were placed unidirectionally. If the breakpoint region was longer than the typical median reads obtained from ONT, we added extra probes approximately every 30 kb, guiding the sequencing in the same direction. See Supplementary Table S1 for detailed information about the Lymphoid Cas9 panel.

### Adaptive sampling target regions

AS for relevant runs was enabled by providing the hg38 reference FASTA file and a BED file containing the target regions of interest (ROIs). According to the ONT technical recommendation, we selected the broader pan-cancer panel to reach approximately 5% of genome coverage, providing optimal performance. We compiled ROIs from commercial panels like TruSight Oncology 500 panel (Illumina), xGen Pan-cancer panel (IDT), Oncomine Comprehensive panel (Thermo Fisher), or Nonacus tumor panel (Nonacus) to obtain a large selection of genes relevant in tumorigenesis across different cancer types. The final BED file (provided in Suppl. Table S2) contains 1320 genes, covering approximately 165 Mb. The genomic coordinates were downloaded from the EMBL Biomart interface [17] and comprise the whole coding sequence of the gene, including all exons and intronic regions flanked by 10 kb buffer from each side. To validate the AS panel, we used the EPI2ME bed-bugs tool [18] to check for any errors.

### Preparation of enriched libraries

Cas9 libraries were prepared using the IDT Crispr-Cas9 in vitro cleavage protocol and ONT ligation sequencing gDNA (SQK-LSK110) protocol with three ug of DNA as an input. For the Cas9-multiplexed library, we proceeded according to the unsupported ONT protocol that combines the Cas9 chemistry with the LSK-NBD-114 protocol. For the AS libraries, we used one ug of each sample according to the singleplex LSK-110 kit protocol or LSK-NBD114 kit protocol for barcoded samples. Detailed procedures are provided in Supplementary Methods.

### Sequencing and data analysis

We used 200–300 ng of final libraries for loading on the MinION R9.4.1 or PromethION R10.4.1 flowcell. Sequencing runs were set up in MinKNOW sequencing software for 72-h runs, first loaded with a half volume of the prepared sequencing library. After 24–48 h, the run was paused and washed with the ONT Wash kit EXP-WSH004, primed and loaded repeatedly with the remaining library volume.

Sequenced data were processed in real-time using the Dorado basecaller current model (v4.3.0) set to high-accuracy (HAC) base calling. The obtained FASTQ files were checked for quality and length of the reads using the Nanoplot tool [19]. Subsequently, reads were mapped using the minimap2 alignment program [20] and the human reference hg38. Coverage of targeted genes was evaluated using samtools [21]. Programs NanoSV v1.2.4 [22] and Sniffles v2.06 [23] were used to detect structural variants. We used the IGV software [24] and Figeno [25] to visualize and manually check identified translocation breakpoints. Inputs to Figeno were prepared using a structural variant predictor DELLY [26].

## Results

### Cas9 enrichment provides superior coverage of ROIs

**Fig. 1:**
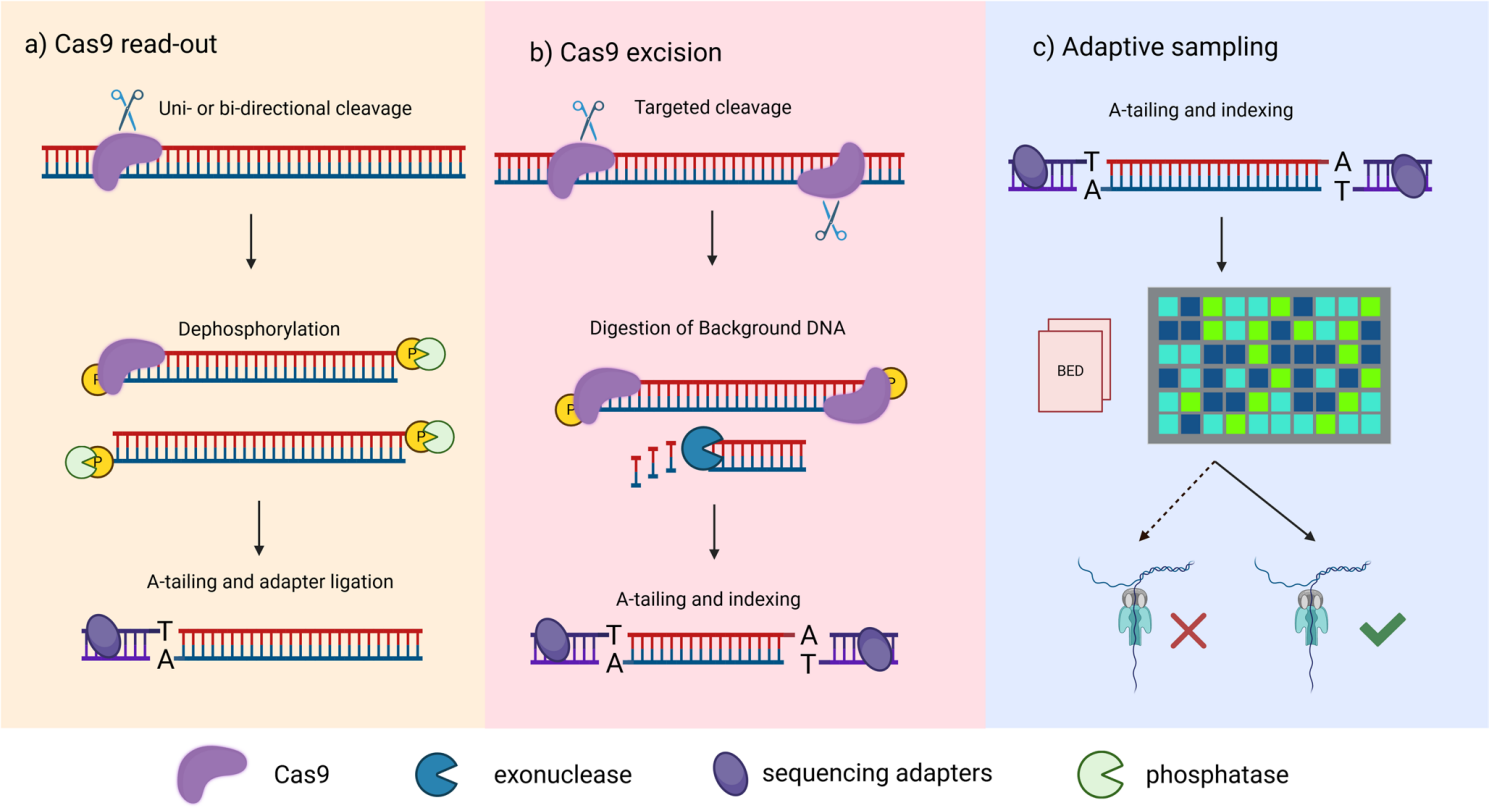
Three different strategies for target enrichment. Regions of interest can be targeted with a) one-sided cleavage of the target region for a unidirectional sequencing, b) cleavage of the ROI from both sides, digestion of background DNA by exonucleases, and adapter ligation (barcoding can be optionally used), and c) adaptive sampling for real-time ROI sequencing with barcoding option. Created in Biorender.com.

CRISPR/Cas9 enrichment strategy uses the Cas9 enzyme to selectively cleave the DNA in the target location of a guide RNA probe (Table 1). In this type of experiment, a breakpoint can be either captured bidirectionally, in the case of probes flanking the expected translocation, or unidirectionally from either side of the cleavage (Fig. 1A). However, samples must be evaluated individually as this approach does not support indexing 2.

**Table 1:** Target regions for Cas9 enrichment approach detecting the most common chromosomal translocations.

Dg	Translocation	Target gene	Chr	Target region (GRCh38)	DNA strand	Cut approach
DLBCL	t(13;14)	BCL6	chr3	187739899–187789899	+	excision
		IGHJ	chr14	105812071–105862071	-	
		IGHM	chr14	105818313–105868313	-	
		IGHC	chr14	105582986–105632986	-	
		IGHC	chr14	105603768–105653768	-	
		IGHC	chr14	105702880–105752880	-	
		IGHC	chr14	105730923–105780923	-	
MCL	t(11;14)	CCND1	chr11	69487659–69537659	-	excision
		IGHJ	chr14	105862708–105912708	+	
FL	t(14;18)	BCL2	chr18	63058339–63108339	-	excision
		BCL2	chr18	63077584–63127584	-	
		IGHJ	chr14	105862708–105912708	+	
BL	t(8;14)	MYC	chr8	127688289–127738289	-	excision
		IGHC	chr14	105625076–105675076	+	
		IGHC	chr14	105770687–105820687	+	
		IGHC	chr14	105855609–105905609	+	
		IGHC	chr14	105643803–105693803	+	
		IGHC	chr14	105742081–105792081	+	
B-ALL	t(11;?)	KMT2A	chr11	118481770–118531770	+	read-out
		KMT2A	chr11	118481863–118531863	+	
	t(9;22)	BCR	chr22	233221201–233271201	-	
		BCR	chr22	23237763–23287763	+	
		BCR	chr22	23216926–23266926	+	
	t(14;?)	IGHC	chr14	105625076–105675076	-	
		IGHC	chr14	105770687–105820687	-	
		IGHC	chr14	105855609–105905609	-	
		IGHC	chr14	105643803–105693803	-	
		IGHC	chr14	105742081–105792081	-	
	t(12;21)	RUNX1	chr21	34859486–34909486	+	excision
		ETV6	chr12	11869511–11919511	+	
		ETV6	chr12	11876674–11926674	+	

*Dg* diagnosis, *Chr* chromosome, *DLBCL* diffuse large B-cell lymphoma, *MCL* mantle cell lymphoma, *FL* follicular lymphoma, *BL* burkitt lymphoma, *B-ALL* B-cell acute lymphoblastic leukemia

**Fig. 2:**
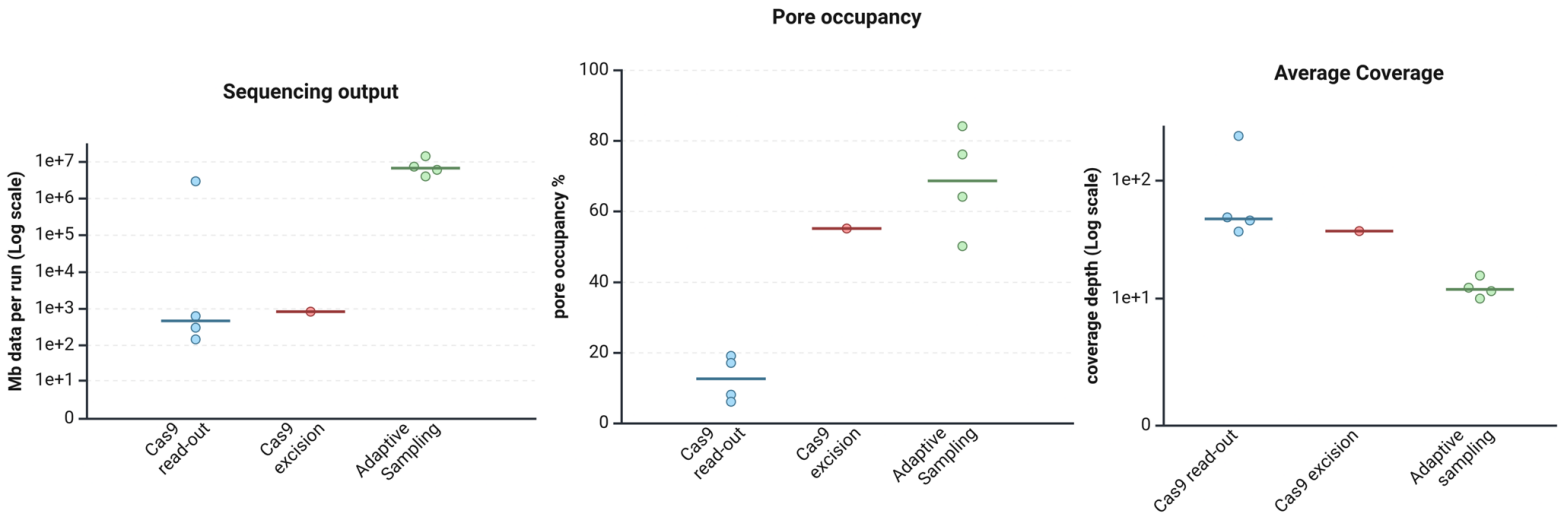
The comparison of sequencing parameters among three enrichment strategies. Created in Biorender.com.

**Fig. 3:**
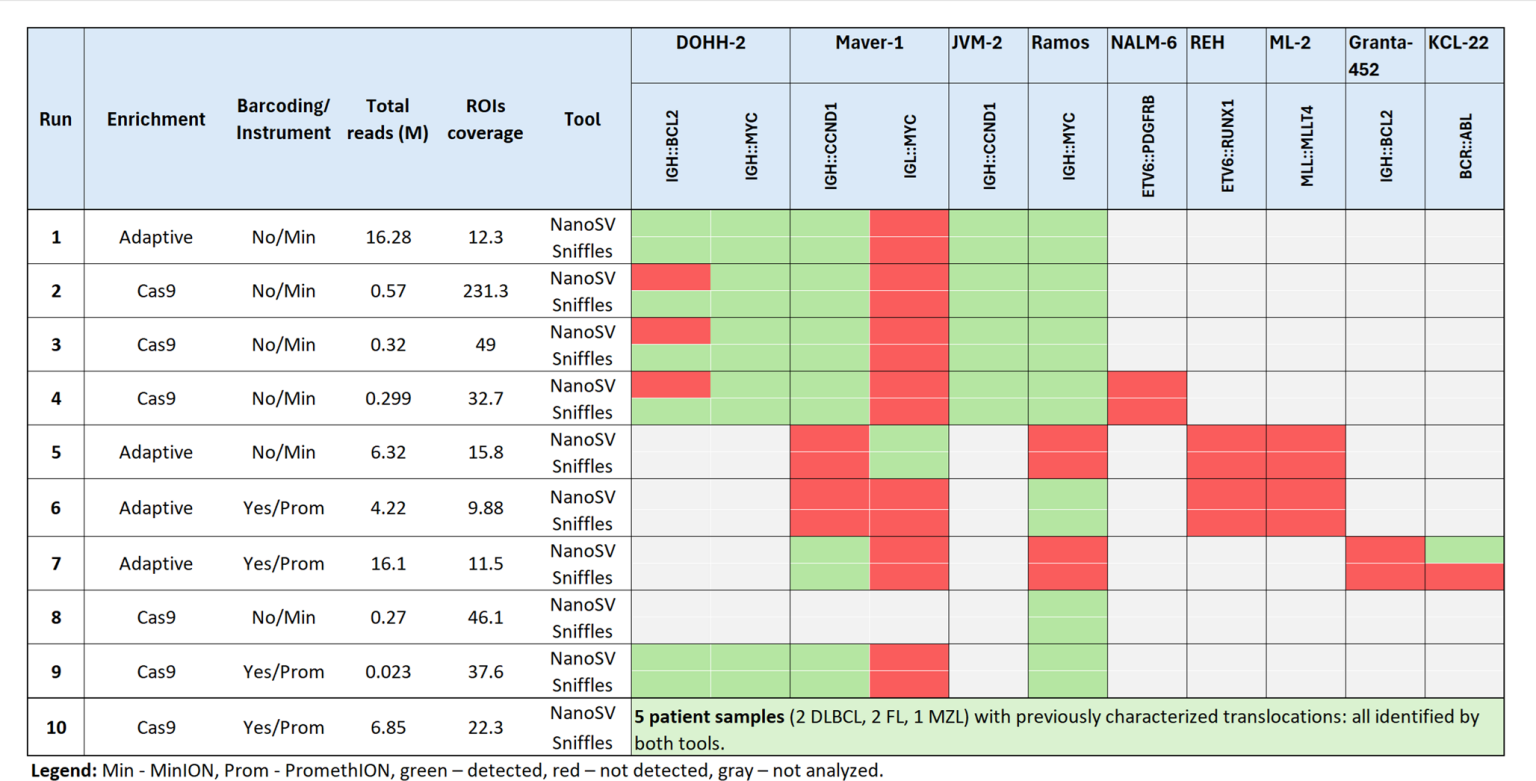
Ability of various enrichment and analytical approaches to detect chromosomal translocations in analyzed cell lines and clinical lymphoma samples. For exact breakpoint location, see Supplementary Table S4 and S5.

We performed four sequencing runs (#2, #3, #4, #8 as indicated in Fig. 3) with this setup using the MinION R9.4.1 flowcell and LSK-110 kit (Suppl. Table S3). Cas9 sequencing runs generated an average of 364,750 reads and 977 Mb sequencing data per flowcell. The sequencing run #2 outperformed other Cas9 runs with 2,86 GB of sequencing data, higher pore occupancy (PO = 19%), and target coverage. The average PO across experiments was 13%. Coverage across ROIs was sufficient to evaluate breakpoints, reaching 231 × average coverage depth in run #2 and 33x-49 × in other experiments. The run output characteristics are visualized in Fig. 2.

First three experiments with Cas9 (run#2, run#3, run#4) included the DNA from cell lines DOHH-2, Maver-1, JVM-2, and Ramos, mixed in equimolar amounts, carrying the sole or multiple translocations *IGH::BCL2*, *IGH::MYC*, *IGH::CCND1*, and *IGL::MYC*. Using the Sniffles tool for SV detection, we identified all the breakpoints (visualized in Suppl. Figure S1), except for the Maver-1 *IGL::MYC* translocation, likely because the Cas9 probes were designed to target only the *MYC* and not the IGL partner. Analyzing the same data with the NanoSV tool, *IGH::BCL2* translocation attributed to the DOHH-2 cell line was missed, albeit with satisfactory region coverage. Sequencing run #4 also included the NALM-6 cell line, but in this case, both tools failed to detect the translocation *ETV6::PDGFRB* present in this cell line due to insufficient coverage. In run #8, the Ramos cell line was used as an input, and we successfully detected the *IGH::MYC* breakpoint with both bioinformatic tools (Fig. 3, Suppl. Table S4).

### Optimized Cas9 multiplexing enhances efficiency without compromising sensitivity

To enhance the applicability and efficiency of our Cas9 enrichment method, we adapted the protocol to be compatible with the latest ONT chemistry, specifically the barcoding kit NBD-114 and PROM-114 flow cells. This adaptation implemented sample multiplexing and a dual excision approach (Fig. 1B), which allows for more precise targeting of common translocations. The sequencing run #9 achieved an average coverage of 37.6x, which aligns with the previous, non-multiplexed runs. We detected four out of five expected translocations (Fig. 3, Suppl. Table S4). Again, we could not detect the translocation *IGL::MYC* due to one-sided cleavage. Other translocations were flanked by Cas9 probes from both sides. By using a multiplexed Cas9 protocol, we achieved higher sequencing yields (810 Mb) and improved flowcell usage (PO 55%), allowing us to process three distinct samples in a single sequencing run simultaneously.

### Adaptive sampling represents a fast and flexible approach with sufficient sequencing output

We compared the Cas9-based enrichment approach with AS (Fig. 1C), offering dynamic control over the sequencing process. Our comparison focused on two key aspects: enrichment efficiency and sample multiplexing capabilities.

In four runs, we obtained average coverage of 12.4 × across the targets defined in the BED file (run #1, #5, #6, #7 in Suppl. Table S3). Coverage uniformity across the target regions was more even than in the case of Cas9 enrichment. This method has an intrinsically higher off-target rate due to the technical character of enrichment. This leads to higher pore occupancy and sequencing yield.

The first run using AS detected the *IGH::MYC*, *IGH::BCL2*, and *IGH::CCND1* translocations typical for DOHH-2, Maver-1, JVM-2, or Ramos cell lines. Run #5 managed to detect only the Maver-1 *IGL::MYC* translocation but missed other expected translocations. Similar results were obtained from runs #6 and #7, where some translocations could be identified, but this approach frequently missed some genetic lesions in the sample. In run #6, we detected only one (*IGH::MYC* translocation of Ramos cell line) out of five major rearrangements that were present; in run #7, we detected two (*IGH::CCND1* and *BCR::ABL1*) out of five translocations (Fig. 3, Suppl. Table S4). The higher rate of undetected breakpoints with AS is attributed to the generally lower coverage compared to Cas9-based approaches (see illustrative Suppl. Figure S2 and S3).

The manufacturer fully supports sample barcoding in conjunction with AS, using the LSK-NBD114 kit. We used this chemistry to prepare the samples for runs #6 and #7. As opposed to the multiplexing of Cas9, where multiplexing had a beneficial effect on PO and yield, in the case of AS, the flow cells were already fully occupied even when sequencing a single sample.

### Proposed decision model applicable for clinical use

**Fig. 4:**
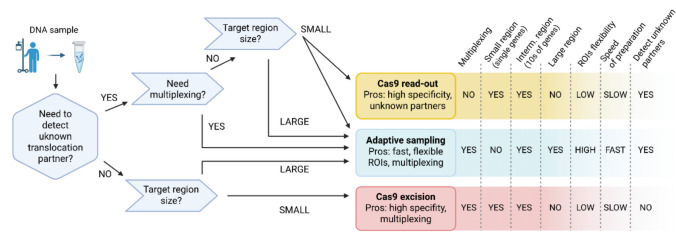
A decision algorithm to select an appropriate enrichment strategy.

After comparing the performance of the three enrichment strategies, we proposed a practical decision algorithm to guide long-read sequencing in both experimental and clinical settings for translocation detection. Key decision points include the size and complexity of ROIs, expected translocation partners (canonical vs. unknown), design flexibility, the need for sample multiplexing, and turnaround time (Fig. 4). From an economic perspective, it is also essential to consider the primary purpose of sequencing (discovery-driven vs. confirmatory), project character (research vs. diagnostic), and how to balance the urgency with cost-effectiveness. Notably, the upfront cost of Cas9 probes is considerable and may influence the decision-making process. For illustrative purposes, we performed a pilot experiment using the Cas9 excision approach to enable rapid and targeted detection of common translocations in five diagnostic lymphoma patients. All expected translocations previously identified by short-read sequencing and/or optical genome mapping were successfully detected (Fig. 3, Suppl. Table 5). In contrast to the Cas9 approach, AS can offer a flexible and cost-efficient alternative for exploratory research without custom probe synthesis. Ultimately, while our decision framework offers a useful guide, laboratories must still rigorously assess and validate their chosen strategy to ensure reliable outcomes.

## Discussion

Long-read sequencing technologies have opened new opportunities for targeted analysis of structural variations [27]. One of the key challenges in detecting translocations lies in their high genomic heterogeneity. For instance, the *KMT2A* has over 80 known translocation partners, and breakpoints upstream of the *MYC* may span several hundred kilobases [28, 29]. This complexity poses significant limitations for conventional detection methods like targeted PCR, which require prior knowledge of breakpoint locations and often involve labor-intensive workflows. Similarly, FISH provides limited information about the precise location and structure of rearrangements. Long-read targeted sequencing or, alternatively, optical genome mapping offers a way to overcome these limitations by enabling the capture of extended genomic regions and complex events. This allows not only the detection of canonical translocation partners, but also the discovery of novel or atypical breakpoints [30, 31].

The Lymphoid Cas9 panel covers clinically relevant lymphoid translocations [16]. Specific genomic regions are targeted using flanking guide RNAs that ensure efficient enrichment. The excision strategy, where both sides of a known target region are cut, demonstrates higher reliability and coverage. This approach is well-suited not only for individual samples but also supports multiplexing, making it more cost-effective and suitable for potential diagnostic applications. However, it fails to detect non-canonical or promiscuous partners, which may involve unexpected regions outside the flanked loci. In contrast to a study by Scholz et al. [32], where multiplexing did not achieve the desired coverage of 50 × for repeat expansion analysis (using R10 MinION flow cell), our results indicate that multiplexing can be successfully implemented for translocation detection. We attribute this improvement to the use of the PromethION R9.4.1 flow cell, which supports higher throughput. Additionally, we confirm that the use of proteinase K did not increase sequencing output or pore occupancy in our internal experiment (data not shown).

The alternative cut-and-read out strategy, which employs single-directional guides adjacent to translocation hotspots, enables the detection of unknown translocation partners. The use of bidirectional probes, as in the excision approach but without exonuclease treatment, facilitates the unexpected partner identification by capturing rearranged fragments from both directions. This approach's major drawback is the lack of an indexing scheme to distinguish individual samples. Although Stangl et al. [12] proposed a post-sequencing method utilizing PCR, this adds complexity and extends turnaround time, limiting its use in clinical workflows. The trade-off between sample multiplexing and broad detection range underscores the careful experimental setup to prioritize high-throughput analysis or sensitive detection of diverse genomic rearrangements.

Our results demonstrate that AS provides greater flexibility for sample multiplexing, albeit with reduced enrichment efficiency compared with Cas9-mediated methods, which consistently achieve higher on-target rates. This difference likely stems from the fundamental principle of AS, which may result in the loss of some on-target reads, thereby reducing overall enrichment performance. This AS limitation for clinical settings has also been recognized in a recent study by Kato et al. [14], which suggested that long-read sequencing may be best used as a complementary method alongside short-read sequencing. Nonetheless, AS retains notable advantages in specific research or clinical scenarios [33]. Its ability to dynamically adjust targeting parameters between sequencing runs and to accommodate a larger number of multiplexed samples makes it particularly suitable for studies prioritizing high sample throughput.

This study has several limitations that should be acknowledged. The sequencing coverage of AS runs was relatively low, which restricted the ability to detect certain breakpoints. Our experiments were conducted on established cell lines, followed by a pilot run on primary patient samples. While this allowed for controlled benchmarking of enrichment strategies, it may not fully capture the complexity of clinical samples, such as tumor heterogeneity, subclonal events, or sample purity. Therefore, the strategies described here require further validation and cannot yet replace conventional diagnostic methods for lymphoid malignancies, though they can serve as a valuable supplement.

From a clinical perspective, long-read sequencing offers unique advantages for detecting and characterizing structural variants in hemato-oncology. Its ability to span large genomic regions and resolve breakpoints at the nucleotide level makes it particularly valuable in cases with ambiguous or inconclusive results from conventional cytogenetic techniques. Moreover, cryptic or complex rearrangements are more reliably captured with ONT technology [27]. On the other hand, the preference for high molecular weight DNA imposes a constraint on clinical implementation, where FFPE samples are commonly used in diagnostic lymphoma workflows. This material typically yields degraded or fragmented DNA [34].

In conclusion, the evaluated enrichment strategies represent viable options for detecting lymphoma-associated translocations. However, their performance is strongly influenced by the specific experimental or clinical objectives and thus requires careful assessment for each application. Cas9-mediated approaches offer high enrichment efficiency and are well suited for high-confidence diagnostic assays, whereas adaptive sampling provides greater flexibility for high-throughput research settings. Here, we demonstrated the potential of Cas9-excision as a rapid molecular examination to detect canonical translocations in primary lymphoma samples. With further optimization and validation in clinical environments, long-read sequencing could be incorporated into routine diagnostic workflows to refine cases with ambiguous cytogenetic findings.

## Supplementary Information

Below is the link to the electronic supplementary material.

Supplementary file1 (XLSX 75 KB)

Supplementary file2 (DOCX 1222 KB)

Supplementary file3 (DOCX 17 KB)

## Data Availability

Data is available upon request.
