# Supplementary material for: Assessment of long-read strategies for the enrichment of clinically relevant breakpoints in lymphomas: towards a diagnostic implementation
Source: Ann Hematol. 2026 Jan 21;105(2):47. doi: 10.1007/s00277-026-06754-2 (PMC12823746; doi:10.1007/s00277-026-06754-2)

**Supplementary Figures**

**Supplementary Figure S1:** **Successfully detected translocations in the Run 3 (Cas9 read-out approach without multiplexing).** This run provides a clear demonstration of sufficient coverage in target regions and unambiguously mapped reads, allowing for exact breakpoint localization (see Supplementary Table S4). Linked reads are colored with the same color; grey represents unlinked reads. Translocations *IGH::BCL2, IGH::MYC,* and *IGH::CCND1* are shown.


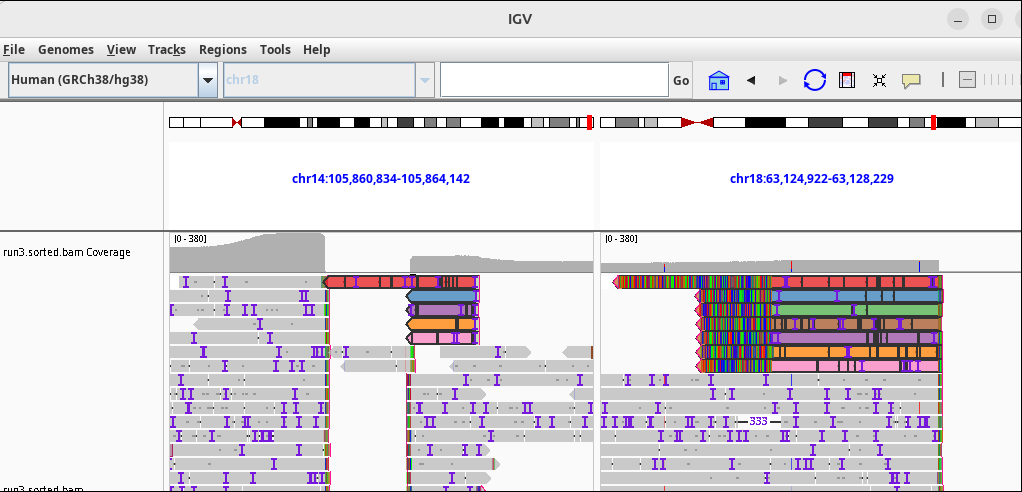


*IGH::BCL2*


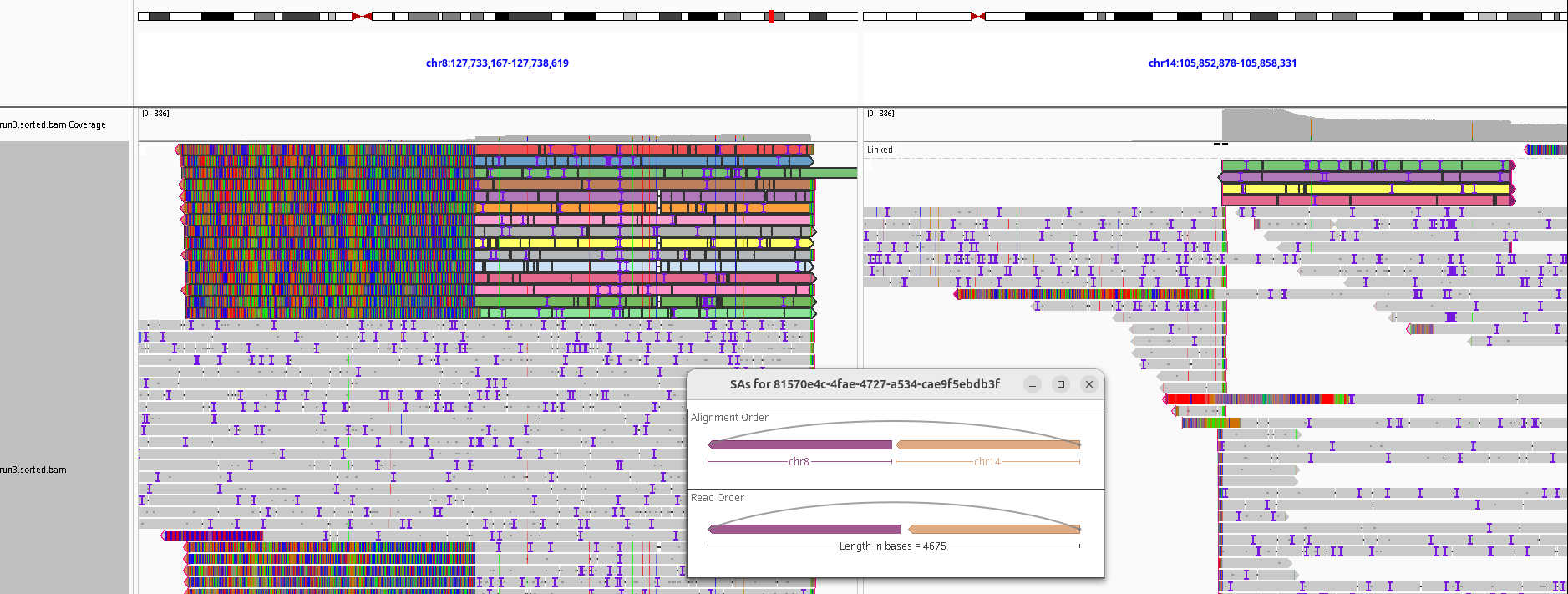


*IGH::MYC*


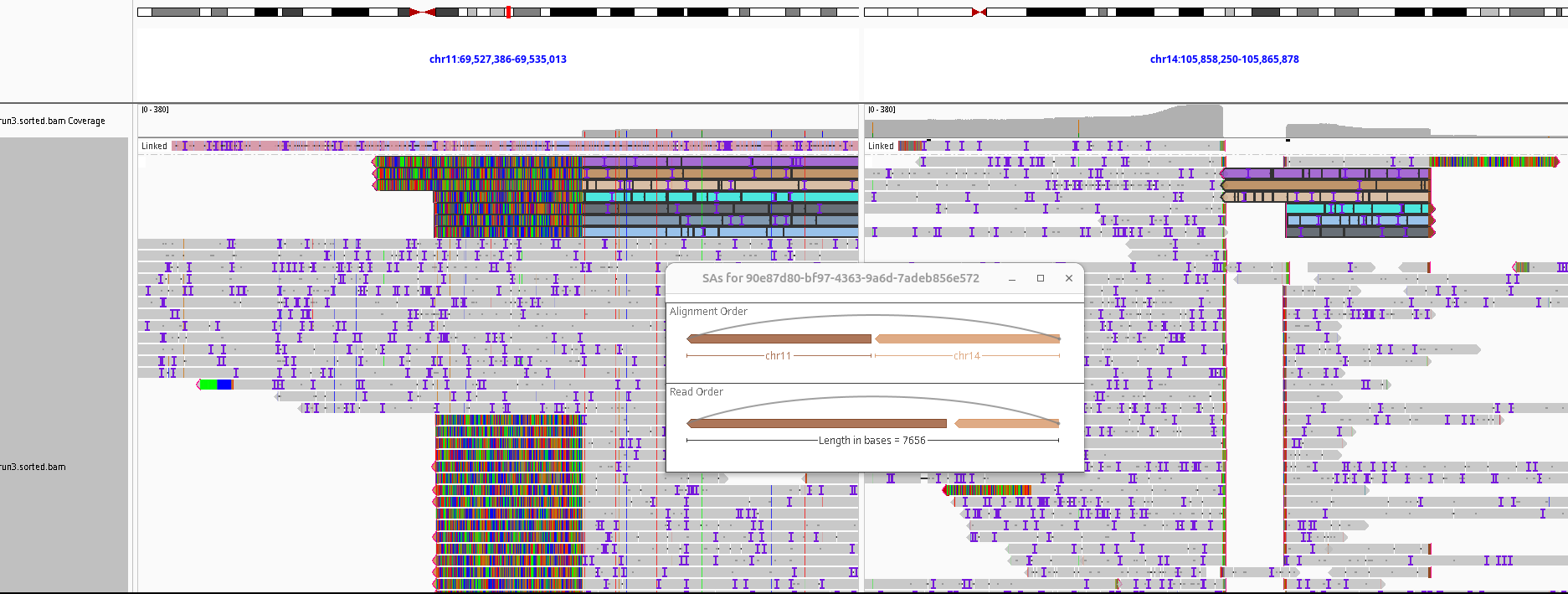


*IGH::CCND1*

**Supplementary Figure S2: Failure to detect translocations in the Run 6 (adaptive sampling with barcoding).** The overall coverage was generally low in this run, hampering the breakpoint detection. (A) An example of the MAVER-1 cell line with the barcode BC13 (in red frame), where no reads supporting the expected *IGH::CCND1* translocation were present; (B) Even the identified *IGH::MYC* translocation in the Ramos cell line (BC11) is supported by only two out of three total reads.


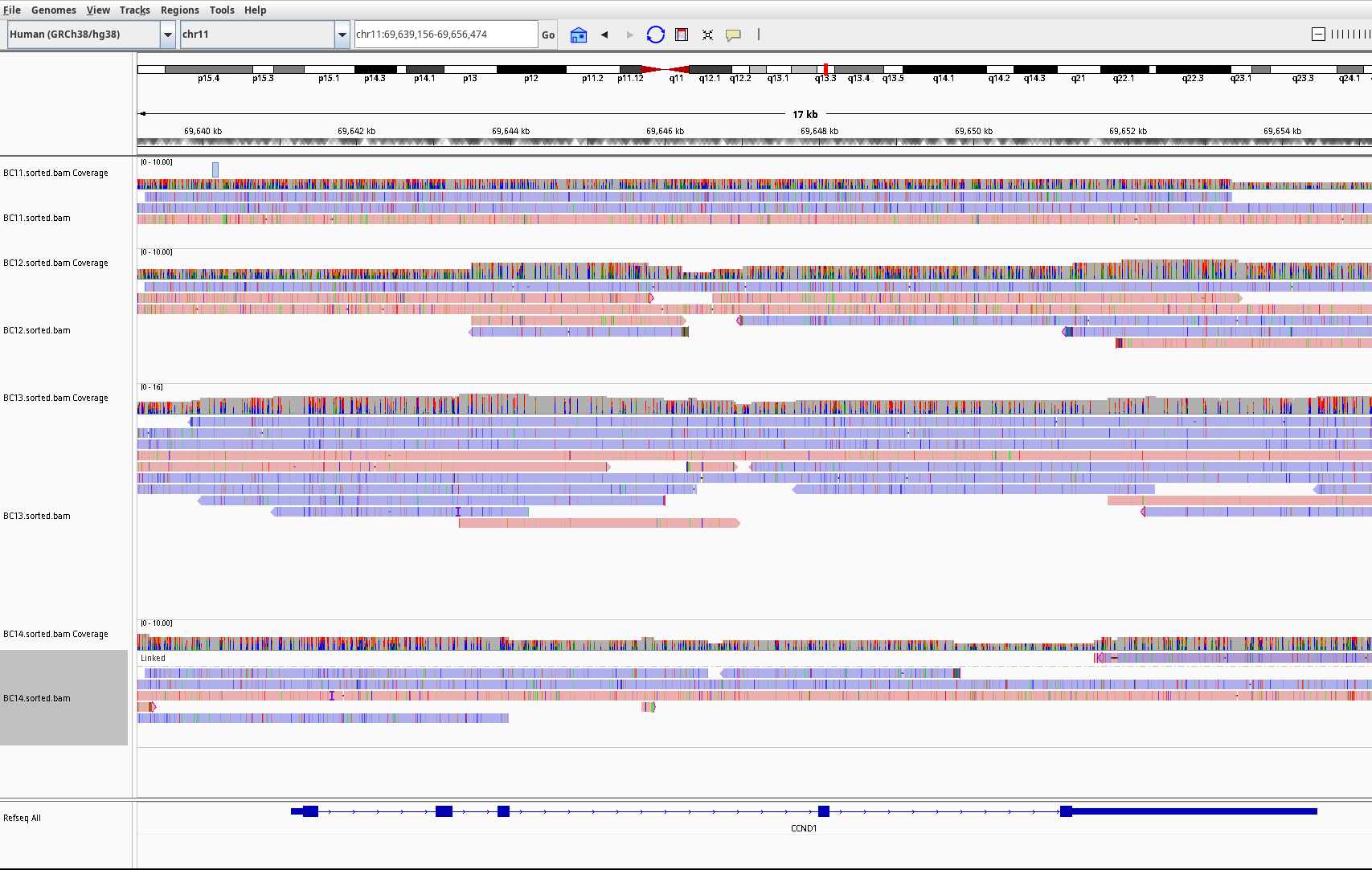


(A)


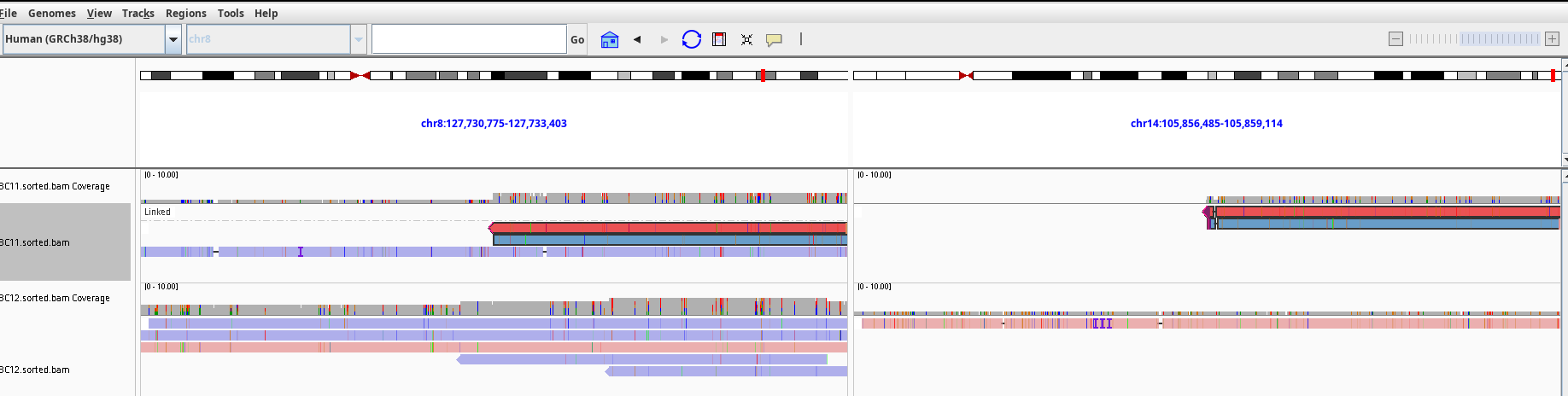


(B) *IGH::MYC*

**Supplementary Figure S3: Uneven coverage across the samples in the Run 7 (adaptive sampling with barcoding).** Although the samples were pooled in an equimolar fashion throughout the experiments, uneven read counts can be observed among samples within a single run. Here is an example of the cell line Granta-452 (BC23, in the red frame), where no reads supporting *IGH::BCL2* translocation were found.


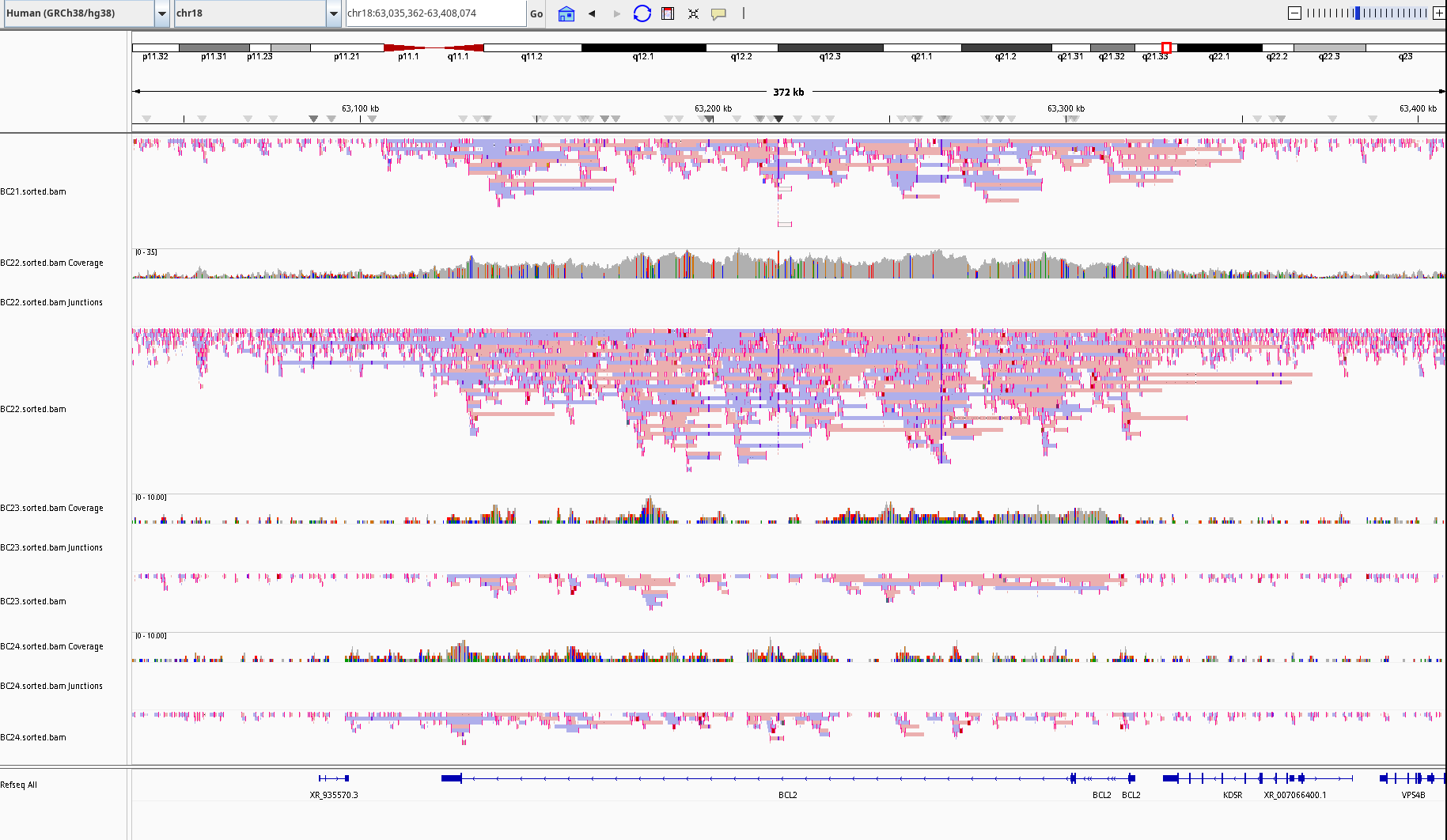

Supplement: Supplementary file 2 — (DOCX 1222 KB) [file 277_2026_6754_MOESM2_ESM.docx]
