# Supplementary material for: Assessment of long-read strategies for the enrichment of clinically relevant breakpoints in lymphomas: towards a diagnostic implementation
Source: Ann Hematol. 2026 Jan 21;105(2):47. doi: 10.1007/s00277-026-06754-2 (PMC12823746; doi:10.1007/s00277-026-06754-2)
Supplement: Supplementary file 3 — (DOCX 17 KB) [file 277_2026_6754_MOESM3_ESM.docx]

**Supplementary Methods**

*Experimental workflow and technical challenges*

To facilitate reader orientation and clarity, we provide a concise overview of our project workflow and experimental development. Our primary goal was to systematically evaluate manufacturer-recommended enrichment strategies that had not yet been assessed for clinically relevant lymphoma breakpoints. To establish a controlled experimental framework, we initially employed well-characterized cell lines to optimize the sequencing workflow before extending our analyses to clinical samples. During this evaluation, it became apparent that each enrichment strategy exhibited specific technical limitations, prompting a study focus on these methodological aspects.

The study primarily utilized established lymphoma and leukemia cell lines available in our laboratory at Masaryk University. The first three sequencing runs were performed using four readily accessible lymphoma cell lines (DOHH-2, MAVER-1, JVM-2, and Ramos). To expand the spectrum of detectable translocations, additional cell lines (NALM-6, REH, ML-2, GRANTA-452, KCL-22) were obtained through collaboration with the University Hospital Brno, which was particularly relevant for Cas9-based experiments. Run 8 included only the Ramos cell line, as the specific aim was to assess the effect of proteinase K treatment on sequencing yield; however, this intervention did not improve the results. In run 9, we returned to the original four cell lines to evaluate a Cas9 multiplexing approach intended for subsequent application to clinical mature B-cell lymphoma samples.

A challenge encountered throughout the study was low and/or uneven sequencing coverage, which in some cases hindered reliable breakpoint detection. Although initial experiments targeted a limited set of genes and translocation partners, we subsequently expanded the regions of interest (ROIs) for adaptive sampling and utilized a PromethION flow cell to increase overall data yield. For Cas9-based enrichment, constructing a large panel is economically impractical, whereas smaller panels generate low library molarity and insufficient pore occupancy. Multiplexing was explored as a potential solution; however, it introduced additional constraints. Cas9 read-out does not inherently support sample indexing and requires additional PCR steps for sample discrimination. In contrast, Cas9 excision allows for multiplexing, but only for known translocation partners. Adaptive sampling theoretically enables broader multiplexing, but our lymphoid-focused panel was too small to achieve efficient enrichment. Even when processing 4–5 samples, per-sample coverage remained low, and including more samples would likely compromise turnaround times in a diagnostic context.

Our findings provide practical guidance for laboratories implementing targeted Nanopore sequencing, illustrating the advantages and limitations of each enrichment strategy and highlighting the need for method-specific optimization by individual users. The decision algorithm we propose reflects these trade-offs and is intended to support strategy selection for structural variant detection. It should, however, be regarded as a conceptual framework requiring validation for each specific application.

*Cas9 library preparation*
Cas9 ribonucleoprotein (RNP) complex was prepared with crRNA and tracrRNA in nuclease-free duplex buffer, incubated for 5 min at 95 °C, and cooled to room temperature (RT). Guide RNA was then mixed with Alt-R S.p. Cas9 enzyme (IDT) in PBS and incubated for 15 minutes at RT. Meanwhile, 3 μg of input DNA were incubated with CIP calf intestinal phosphatase (NEB) in 1x NEB CutSmart buffer at 37°C for 10 minutes and 80 °C for 2 minutes. Dephosphorylated DNA was then mixed with loaded Cas9 RNP complex, 10 mM dATP, and NEB Taq polymerase and incubated for 30 minutes at 37 °C and 5 minutes at 72 °C. Finally, ONT adapters were ligated using the NEBNext Quick T4 ligase. The final library was quantified using the Quantus fluorometer (Promega, WI, USA) and sequenced on a MinION R9.4.1 flow cell.

*Cas9 multiplexing library preparation*

For the Cas9-multiplexed library, we started again with 3 μg of isolated DNA and prepared the library according to the unsupported ONT protocol that follows the general workflow in the paragraph above, with modifications at the end of the protocol.

Cas9 ribonucleoprotein (RNP) complex was prepared with crRNA and tracrRNA in nuclease-free duplex buffer, incubated for 5 min at 95 °C, and cooled to room temperature (RT). Guide RNA was then mixed with Alt-R S.p. Cas9 enzyme (IDT) in PBS and incubated for 15 minutes at RT. Meanwhile, 3 μg of input DNA were incubated with CIP calf intestinal phosphatase (NEB) in 1x NEB CutSmart buffer at 37°C for 10 minutes and 80 °C for 2 minutes. Dephosphorylated DNA was mixed with the loaded Cas9 RNP complex, 10 mM dATP, and NEB Taq polymerase and incubated for 30 minutes at 37 °C and 5 minutes at 72 °C. After adding Cas9 RNP complex, the sample was treated with a mix of Lambda exonuclease and Exonuclease I to digest background DNA. Remaining fragments were A-tailed using the NEB Taq polymerase and ligated with native barcodes from the LSK-NBD114 kit. Finally, sequencing adapters were ligated with NEBNext Quick Ligation Reaction Module and prepared for the sequencing according to the manufacturer’s protocol. The final library was quantified using the Quantus fluorometer (Promega, WI, USA) and sequenced on PromethION R10.4.1 flow cell.

*Adaptive sampling library preparation*

For the adaptive sampling protocol, we used the LSK-110 kit from Oxford Nanopore and additional chemistry from New England Biolabs, as listed in the official protocol. Briefly, 1000 ng of DNA was used as input for library preparation. After the end-repair and A-tailing steps, DNA was purified with SPRI select beads (Beckman Coulter). Adapter with motor protein was ligated onto the A-tailed ends and purified, using the LFB buffer for the washing steps.

For the multiplexed AS libraries, we used one μg of each sample according to the protocol instructions. The protocol for the LSK-NBD114 kit was followed as described above, with the modification of first ligating barcoded indexes using the NEB Blunt/TA ligase (M0367S, NEB) after the A-tailing step and then following with the ligation of the native adapter.

The final libraries were quantified using the Quantus fluorometer (Promega, WI, USA) and sequenced on MinION R9.4.1 and PromethION R10.4.1 flow cells.
